# Supplementary material for: Mast cells link immune sensing to antigen-avoidance behaviour
Source: Nature. 2023 Jul 12;620(7974):634–42. doi: 10.1038/s41586-023-06188-0 (PMC10432277; doi:10.1038/s41586-023-06188-0)
Supplement: Supplementary file 2 — Reporting Summary [file 41586_2023_6188_MOESM2_ESM.pdf]

nature portfolio

Corresponding author(s): Thomas Plum
Hans-Reimer Rodewald

Last updated by author(s): April 13, 2023

Reporting Summary

Nature Portfolio wishes to improve the reproducibility of the work that we publish. This form provides structure for consistency and transparency in reporting. For further information on Nature Portfolio policies, see our Editorial Policies and the Editorial Policy Checklist.

Statistics

For all statistical analyses, confirm that the following items are present in the figure legend, table legend, main text, or Methods section.

n/a	Confirmed	
☐	☒	The exact sample size (*n*) for each experimental group/condition, given as a discrete number and unit of measurement
☐	☒	A statement on whether measurements were taken from distinct samples or whether the same sample was measured repeatedly
☐	☒	The statistical test(s) used AND whether they are one- or two-sided<br>*Only common tests should be described solely by name; describe more complex techniques in the Methods section.*
☒	☐	A description of all covariates tested
☐	☒	A description of any assumptions or corrections, such as tests of normality and adjustment for multiple comparisons
☐	☒	A full description of the statistical parameters including central tendency (e.g. means) or other basic estimates (e.g. regression coefficient) AND variation (e.g. standard deviation) or associated estimates of uncertainty (e.g. confidence intervals)
☐	☒	For null hypothesis testing, the test statistic (e.g. *F*, *t*, *r*) with confidence intervals, effect sizes, degrees of freedom and *P* value noted<br>*Give P values as exact values whenever suitable.*
☒	☐	For Bayesian analysis, information on the choice of priors and Markov chain Monte Carlo settings
☒	☐	For hierarchical and complex designs, identification of the appropriate level for tests and full reporting of outcomes
☒	☐	Estimates of effect sizes (e.g. Cohen's *d*, Pearson's *r*), indicating how they were calculated

Our web collection on statistics for biologists contains articles on many of the points above.

Software and code

Policy information about availability of computer code

Data collection	BD FACS Diva (v.6.1.2); IntelliCage Plus software (v.3.2.3); Sygnis Tracker software (v.4.1.4); LABORAS software (v.2.6)
Data analysis	Graphpad Prism (v.9.0); FlowJo (v.10.7.1); STAR aligner (v.2.5.2b); subread package (v.1.5.1); DESeq2 (v.1.38.3); clusterProfiler (v.3.1.6); The custom RNA-Seq analysis pipeline has been made publicly available via: https://github.com/robinthiele/AAPIA

For manuscripts utilizing custom algorithms or software that are central to the research but not yet described in published literature, software must be made available to editors and reviewers. We strongly encourage code deposition in a community repository (e.g. GitHub). See the Nature Portfolio guidelines for submitting code & software for further information.

Data

Policy information about availability of data

All manuscripts must include a data availability statement. This statement should provide the following information, where applicable:

- Accession codes, unique identifiers, or web links for publicly available datasets
- A description of any restrictions on data availability
- For clinical datasets or third party data, please ensure that the statement adheres to our policy

The RNA-Seq data, deposited in NCBI's Gene Expression Omnibus, are accessible through GEO Series accession number GSE225054 (https://www.ncbi.nlm.nih.gov/geo/query/acc.cgi?acc=GSE225054). There are no restrictions.

March 2021

Human research participants

Policy information about studies involving human research participants and Sex and Gender in Research.

Reporting on sex and gender	n/a
Population characteristics	n/a
Recruitment	n/a
Ethics oversight	n/a

Note that full information on the approval of the study protocol must also be provided in the manuscript.

Field-specific reporting

Please select the one below that is the best fit for your research. If you are not sure, read the appropriate sections before making your selection.

☒ Life sciences ☐ Behavioural & social sciences ☐ Ecological, evolutionary & environmental sciences

For a reference copy of the document with all sections, see nature.com/documents/nr-reporting-summary-flat.pdf

Life sciences study design

All studies must disclose on these points even when the disclosure is negative.

Sample size

Sample sizes for antigen-avoidance experiments were calculated based on the variability in the data in ref. 4. For other experiments, sample sizes were chosen based on prior experience and pilot experiments for testing statistically significant differences between conditions. In compliance with the regulatory guidelines, a minimal number of animals for statistically significant data was used.

Data exclusions

Due to loss of transponder signal over several days, one mouse was excluded from the IntelliCage analysis of mast cell-deficient BALB/c mice (Fig. 1).

Replication

The main findings were reliably reproduced in independent experiments. All attempts at replication were successful. In the case where one experiment was done the data allowed significant conclusions on differences between genotypes and conditions.
Numbers of experiments:
Data in Fig. 1 are derived from two independent experiments (total number of 51 mice).
Data in Fig. 2a-e are derived from one experiment (total number of 30 mice)
Data in Fig. 2f-i are derived from one experiment (total number of 13 mice)
Data in Fig. 2 j-l are derived from two experiments (total number of 13 mice).
Data in Fig. 3 are derived from two experiments (total number of 89 mice).
Data in Fig. 4b,c are derived from two experiments (total number of 16 mice).
Data in Fig. 4e are derived from one experiment (total number of 30 mice).
Data in Fig. 4f are derived from two experiments (total number of 66 mice).
Data in Ext. Data Fig. 1 are derived from one experiment (total number of 30 mice).
Data in Ext. Data Fig. 2a,b,d-h are derived from two experiments (total number of 48 mice).
Data in Ext. Data Fig. 3a-i are derived from three experiments (total number of 62 mice).
Data in Ext. Data Fig. 3k-m are derived from two experiments (total number of 19 mice).
Data in Ext. Data Fig. 4, 5, 6 are derived from two experiments (total number of 89 mice).
Data in Ext. Data Fig. 7 are derived from two experiments (total number of 30 mice).
Data in Ext. Data Fig. 8a are derived from three experiments (total number of 42 mice).
Data in Ext. Data Fig. 8b are derived from two experiments (total number of 34 mice).
Data in Ext. Data Fig. 8c are derived from one experiment (total number of 21 mice).
Data in Ext. Data Fig. 8d are derived from one experiment (total number of 16 mice).
Data in Ext. Data Fig. 8e,f are derived from one experiment (total number of 12 mice).
Data in Ext. Data Fig. 8g are derived from four experiments (total number of 59 mice).
Data in Ext. Data Fig. 9 are derived from two experiments (total number of 17 mice).
Data in Ext. Data Fig. 10b-e are derived from two experiments (total number of 10 mice).
Data in Ext. Data Fig. 10f are derived from one experiment (total number of 4 mice).
Data in Ext. Data Fig. 10g,h are derived from two independent experiments (total number of 54 mice).
Data in Ext. Data Fig. 10i,j are derived from one experiment (total number of 28 mice).

Randomization

Mice were genotyped, and prior to the experiments assigned to test and control groups. Because the genotype dictates the group assignment no randomization was possible.

Blinding

Investigators were not blinded in setting up experimental groups, as knowledge of genotypes was essential to plan the studies. Regarding data acquisition, scientists were not blinded to the genotypes and the treatment of the animals, except for automated data analysis, such as in IntelliCage experiments, RNA-Seq experiments, and anxienty and general behavioral assays.

Reporting for specific materials, systems and methods

We require information from authors about some types of materials, experimental systems and methods used in many studies. Here, indicate whether each material, system or method listed is relevant to your study. If you are not sure if a list item applies to your research, read the appropriate section before selecting a response.

Materials & experimental systems

n/a	Involved in the study
☐	☒ Antibodies
☒	☐ Eukaryotic cell lines
☒	☐ Palaeontology and archaeology
☐	☒ Animals and other organisms
☒	☐ Clinical data
☒	☐ Dual use research of concern

Methods

n/a	Involved in the study
☒	☐ ChIP-seq
☐	☒ Flow cytometry
☒	☐ MRI-based neuroimaging

Antibodies

Antibodies used

B220 FITC (RA3-6B2, BD Pharmingen, Cat. #553087) 1:50
CD3 BV421 (17A2, Biolegend, Cat. #100228) 1:200
CD3 FITC (17A2, BD Pharmingen, Cat. #555274) 1:50
CD3 Pe-Cy7 (145-2C11, BD Pharmingen, Cat. #552774) 1:25
CD11b PerCP-Cy5.5 (M1/70, eBioscience, Cat. #45-0112-82) 1:400
CD11b BV421 (M1/70, Biolegend, Cat. #101251) 1:400
CD11b PE-Cy-7 (M1/70, eBioscience, Cat. #25-0112) 1:400
CD11c BV421 (N418, Biolegend, Cat. #117330) 1:100
CD16/32 unconjugated (93, Biolegend, Cat. #101301) 10 μg/mL
CD19 BV421 (6D5, Biolegend, Cat. #115537) 1:400
CD19 APC (1D3, BD Pharmingen, Cat. #550992) 1:400
CD45 BV421 (30-F11, Biolegend, Cat. #103133) 1:400
CD45 BV785 (30-F11, Biolegend, Cat. #103149) 1:400
CD49b APC (DX5, BD Pharmingen, Cat. #560628) 1:100
CD90.2 APC-Cy7 (30-H12, Biolegend, Cat. #105328) 1:400
CD117 PE (2B8, eBioscience, Cat. #12-1171) 1:800
CD117 BV711 (2B8, Biolegend, Cat. #105835) 1:800
CD117 APC (2B8, BD Pharmingen, Cat. #553356) 1:800
FcεRI APC (MAR-1, eBioscience, Cat. #17-5898-82) 1:200
Gr-1 BV421 (RB6-8C5, BioLegend, Cat. #108445) 1:800
Gr-1 BV605 (RB6-8C5, Biolegend, Cat. #108439) 1:200
IgE PE (RME1, Biolegend, Cat. #406907) 1:100
IgE BV786 (RME-1, BD Pharmingen, Cat. #564206) 1:100
IgE BV421 (R35-72, BD Pharmingen, Cat. #564207) 1:100
Ly6G PerCP-Cy5.5 (1A8, BD Pharmingen, Cat. #560602) 1:100
MHCII A700 (M5/114.15.2, eBioscience, Cat. #56-5321-82) 1:100
Siglec-F BV421 (E50-2440, BD Pharmingen, Cat. #565934) 1:100
Siglec-F PE (E50-2440, BD Pharmingen, Cat. #552126) 1:100
Ter119 BV421 (Ter119, Biolegend, Cat. #116234) 1:200
5-HT unconjugated (5HT-H209, Dako, Cat. #M0758) 0.11 μg/mL
mouse-IgG1 PE (RMG1-1, Biolegend, Cat. #406607) 1:100
Anti-OVA IgG1 (L71, Biozol, Cat. #CHX-3013) 1:2000
Anti-OVA IgE (2C6, Invitrogen, Cat. #MA1-80396) 1:2000
Anti-mouse IgG1-HRP (X56, BD Pharmingen, Cat. #559626) 1:2000
Anti-mouse IgE-HRP (23G3, SouthernBiotech, Cat. #1130-01) 1:2000

Validation

Validation statement for each antibody is provided on the manufacturer's website:
B220 FITC https://www.bdbiosciences.com/en-de/products/reagents/flow-cytometry-reagents/research-reagents/single-color-antibodies-ruo/fitc-rat-anti-mouse-cd45r-b220.553087
CD3 BV421 https://www.biolegend.com/en-us/products/brilliant-violet-421-anti-mouse-cd3-antibody-7326
CD3 FITC https://www.bdbiosciences.com/en-de/products/reagents/flow-cytometry-reagents/research-reagents/single-color-antibodies-ruo/fitc-rat-anti-mouse-cd3-molecular-complex.555274
CD3 Pe-Cy7 https://www.citeab.com/antibodies/2414263-552774-bd-pharmingen-pe-cy-7-hamster-anti-mouse-cd3
CD11b PerCP-Cy5.5 https://www.thermofisher.com/antibody/product/CD11b-Antibody-clone-M1-70-Monoclonal/45-0112-82
CD11b BV421 https://www.biolegend.com/en-us/products/brilliant-violet-421-anti-mouse-human-cd11b-antibody-7163
CD11b PE-Cy-7 https://www.thermofisher.com/antibody/product/CD11b-Antibody-clone-M1-70-Monoclonal/25-0112-82
CD11c BV421 https://www.biolegend.com/en-us/products/brilliant-violet-421-anti-mouse-cd11c-antibody-7149
CD16/32 unconjugated https://www.biolegend.com/en-us/products/purified-anti-mouse-cd16-32-antibody-190
CD19 BV421 https://www.biolegend.com/en-us/products/brilliant-violet-421-anti-mouse-cd19-antibody-7160
CD19 APC https://www.bdbiosciences.com/en-ca/products/reagents/flow-cytometry-reagents/research-reagents/single-color-

antibodies-ruo/apc-rat-anti-mouse-cd19.550992
CD45 BV421 https://www.biolegend.com/en-us/products/brilliant-violet-421-anti-mouse-cd45-antibody-7253
CD45 BV785 https://www.biolegend.com/en-us/products/brilliant-violet-785-anti-mouse-cd45-antibody-10636
CD49b APC https://www.bdbiosciences.com/en-de/products/reagents/flow-cytometry-reagents/research-reagents/single-color-antibodies-ruo/apc-rat-anti-mouse-cd49b.560628
CD90.2 APC-Cy7 https://www.biolegend.com/en-us/products/apc-cyanine7-anti-mouse-cd90-2-thy1-2-antibody-6671
CD117 PE https://www.thermofisher.com/antibody/product/CD117-c-Kit-Antibody-clone-2B8-Monoclonal/12-1171-82
CD117 BV711 https://www.biolegend.com/en-us/products/brilliant-violet-711-anti-mouse-cd117-c-kit-antibody-12049
CD117 APC https://www.bdbiosciences.com/en-de/products/reagents/flow-cytometry-reagents/research-reagents/single-color-antibodies-ruo/apc-rat-anti-mouse-cd117.553356
FcεRI APC https://www.thermofisher.com/antibody/product/FceR1-alpha-Antibody-clone-MAR-1-Monoclonal/17-5898-82
Gr-1 BV421 https://www.biolegend.com/en-us/products/brilliant-violet-421-anti-mouse-ly-6g-ly-6c-gr-1-antibody-7201
Gr-1 BV605 https://www.biolegend.com/en-us/products/brilliant-violet-605-anti-mouse-ly-6g-ly-6c-gr-1-antibody-8724
IgE PE https://www.biolegend.com/en-us/products/pe-anti-mouse-ige-3267
IgE BV786 https://www.bdbiosciences.com/en-de/products/reagents/flow-cytometry-reagents/research-reagents/single-color-antibodies-ruo/bv786-rat-anti-mouse-ige.564206
IgE BV421 https://www.bdbiosciences.com/en-de/products/reagents/flow-cytometry-reagents/research-reagents/single-color-antibodies-ruo/bv421-rat-anti-mouse-ige.564207
Ly6G PerCP-Cy5 https://www.bdbiosciences.com/en-de/products/reagents/flow-cytometry-reagents/research-reagents/single-color-antibodies-ruo/percp-cy-5-5-rat-anti-mouse-ly-6g.560602
MHCII A700 https://www.thermofisher.com/antibody/product/MHC-Class-II-I-A-I-E-Antibody-clone-M5-114-15-2-Monoclonal/56-5321-82
Siglec-F BV421 https://www.bdbiosciences.com/en-de/products/reagents/flow-cytometry-reagents/research-reagents/single-color-antibodies-ruo/bv421-rat-anti-mouse-siglec-f.565934
Siglec-F PE https://www.bdbiosciences.com/en-de/products/reagents/flow-cytometry-reagents/research-reagents/single-color-antibodies-ruo/pe-rat-anti-mouse-siglec-f.552126
Ter119 BV421 https://www.biolegend.com/en-us/products/brilliant-violet-421-anti-mouse-ter-119-erythroid-cells-antibody-7259
5-HT unconjugated https://www.agilent.com/en/product/immunohistochemistry/antibodies-controls/primary-antibodies/serotonin-(concentrate)-76521
mouse-IgG1 PE https://www.biolegend.com/en-us/products/pe-anti-mouse-igg1-6494
Anti-OVA IgG1 https://www.biozol.de/de/product/CHX-3013
Anti-OVA IgE https://www.thermofisher.com/antibody/product/Ovalbumin-Antibody-clone-2C6-Monoclonal/MA1-80396
Anti-mouse IgG1-HRP https://www.bdbiosciences.com/en-de/products/reagents/immunoassay-reagents/elisa/hrp-rat-anti-mouse-igg1.559626
Anti-mouse IgE-HRP https://www.southernbiotech.com/rat-anti-mouse-ige-unlb-23g3-1130-01

Animals and other research organisms

Policy information about studies involving animals; ARRIVE guidelines recommended for reporting animal research, and Sex and Gender in Research

Laboratory animals	BALB/c wild type, C57BL/ 6 wild type, BALB/c Cpa3Cre/+, C57BL/6 Cpa3Cre/+, BALB/c Igh-7-/-, BALB/c Mcpt8-Cre, BALB/c Cpa3Y356L,E378A, BALB/c Cpa3-/-, BALB/c Hdc-/-, BALB/c Mcpt6-/-, C57BL/ 6 Nr4a1-GFP, BALB/c x C57BL/6 F1 Nr4a1-GFP, and C57BL/6 Wnt1|GCaMP3 (Wnt1-Cre;R26R-GCaMP3) mice. Adult male and female mice from 6-59 weeks of age were used for the study.
Wild animals	The study did not involve wild animals.
Reporting on sex	Both male and female mice were used.
Field-collected samples	The study did not involve samples collected in the field.
Ethics oversight	All animal experiments were performed in accordance with institutional and governmental regulations. Experiments in Heidelberg were approved by the Regierungspräsidium Karlsruhe, Germany. Experiments in Leuven were approved by the Animal Care and Animal Experiments Committee of the KU Leuven, Leuven, Belgium.

Note that full information on the approval of the study protocol must also be provided in the manuscript.

Flow Cytometry

Plots

Confirm that:

☒ The axis labels state the marker and fluorochrome used (e.g. CD4-FITC).

☒ The axis scales are clearly visible. Include numbers along axes only for bottom left plot of group (a 'group' is an analysis of identical markers).

☒ All plots are contour plots with outliers or pseudocolor plots.

☒ A numerical value for number of cells or percentage (with statistics) is provided.

Methodology

Sample preparation	Gingival single cell suspensions were prepared as previously described67. In brief, the palate and mandible were isolated, and tissues were digested for 1h at 37°C in RPMI supplemented with 10% FCS (Sigma-Aldrich), 0.15 µg DNase I, and 3.2 mg/mL collagenase IV (all enzymes from Sigma-Aldrich). 0.5M EDTA (Roth) was added during the last 5 min, and supernatant was filtered through a 70 µm cell strainer (ThermoFisher). Undigested gingiva tissue was then peeled from palate and mandible and mashed through the same filter to yield the gingiva cell suspension.<br>Tongue single cell suspensions were prepared by finely mincing the tongue and digesting the tissue for three rounds of 15-minutes at 37°C in RPMI supplemented with 0.1 mg/mL Liberase TM (Sigma-Aldrich) and 2.5 µg/mL DNase I (Sigma-Aldrich). After each round of digestion, the cell suspensions were filtered through a 70 µm cell strainer (ThermoFisher), and new enzyme solution was added to the tissue. All fractions were combined to yield the tongue single cell suspension.<br>For isolation of stomach intraepithelial leukocytes, the stomach was cut open and food remnants were removed. Stomachs were incubated for 15 min at 37°C in HBSS supplemented with 20 mM EDTA (Roth) to release the epithelial layers from the connective tissue. The cell suspension was applied onto a spin column (ThermoFisher) packed with 100 µm zirconia beads (Roth). After centrifugation, the flow through was collected yielding the intraepithelial cell suspension containing mucosal stomach mast cells.<br>For preparation of small intestine cell suspensions, small intestines were cut open and food remnants were removed. Intestines were incubated for 15 min at 37°C in HBSS supplemented with 2% FCS (Sigma-Aldrich), 5 mM EDTA (Roth), 1 mM DTT (Merck), and 10 mM HEPES (Life Technologies) to release the epithelial layers from the connective tissue. The cells in the soluble fraction (containing intraepithelial mast cells) were filtered through a 70 µm cell strainer (ThermoFisher). The remaining intestine tissue was washed in PBS and transferred into RPMI supplemented with 2% FCS (Sigma-Aldrich), 20 mM HEPES (Life Technologies), 0.2 mg/mL collagenase IV (Sigma-Aldrich), 0.5 mg/mL Hyaluronidase I (Sigma-Aldrich), and 0.1 mg/mL DNase I (Sigma-Aldrich). Digestion was carried out for 30 min at 37°C and digested tissue was filtered through a 100 µm cell strainer (ThermoFisher) yielding the lamina propria fraction (containing lamina propria mast cells).<br>Blood was drawn by cardiac puncture, followed by red blood cell lysis according to the manufacturer's protocol (RBC Lysis Buffer, BioLegend).
Instrument	LSR Fortessa (BD Bioscience)
Software	BD FACS Diva (v 6.1.2)
Cell population abundance	Cell populations were quantified as absolute numbers (per stomach or per microliter of blood), or frequencies of total live cells (intestine).
Gating strategy	Tongue and gingiva mast cells: live CD45+MHCII−CD11b−CD117+FcεRI+<br>Stomach mast cells: live CD45+CD117+FcεRI/IgE+<br>Intestinal mast cells: live CD45+CD3-CD19-Gr-1-Ter119-Siglec-F-CD117+FcεRI+<br>Basophils: live CD45+CD90.2-CD3- CD11c-Gr-1-Siglec-F-B220-MHCII-CD49b+IgE+<br>Stomach neutrophils: live CD45+CD11b+MHCII-Siglec-F-Ly6G+<br>Small intestine neutrophils: live CD45+CD11b+MHCII-Siglec-F-Gr-1+

☒ Tick this box to confirm that a figure exemplifying the gating strategy is provided in the Supplementary Information.
